# AttentionMNIST: a mouse-click attention tracking dataset for handwritten numeral and alphabet recognition

**DOI:** 10.1038/s41598-023-29880-7

**Published:** 2023-02-27

**Authors:** Murchana Baruah, Bonny Banerjee, Atulya K. Nagar, René Marois

**Affiliations:** 1 Institute for Intelligent Systems, and Department of Electrical & Computer Engineering, University of Memphis, Memphis, TN 38152 USA; 2 School of Mathematics, Computer Science and Engineering, Liverpool Hope University, Hope Park, Liverpool, L16 9JD UK; 3 Department of Psychology, Vanderbilt Vision Research Center, Vanderbilt Brain Institute, Vanderbilt University, Nashville, TN 37240 USA

**Keywords:** Computer science, Scientific data, Human behaviour

## Abstract

Multiple attention-based models that recognize objects via a sequence of glimpses have reported results on handwritten numeral recognition. However, no attention-tracking data for handwritten numeral or alphabet recognition is available. Availability of such data would allow attention-based models to be evaluated in comparison to human performance. We collect mouse-click attention tracking data from 382 participants trying to recognize handwritten numerals and alphabets (upper and lowercase) from images via sequential sampling. Images from benchmark datasets are presented as stimuli. The collected dataset, called AttentionMNIST, consists of a sequence of sample (mouse click) locations, predicted class label(s) at each sampling, and the duration of each sampling. On average, our participants observe only 12.8% of an image for recognition. We propose a baseline model to predict the location and the class(es) a participant will select at the next sampling. When exposed to the same stimuli and experimental conditions as our participants, a highly-cited attention-based reinforcement model falls short of human efficiency.

## Introduction

Machine learning (ML) models that recognize objects via a sequence of glimpses have gained interest in recent years due to their scalability and efficiency. Many of these models, such as^1–7^, have reported experimental results on the benchmark MNIST dataset for handwritten numeral recognition. Unfortunately, no attention tracking data for the MNIST is available. This prevents the evaluation of attention-based models in comparison to human performance.

We fill in that gap by collecting a dataset from adult participants trying to recognize handwritten numerals and alphabets from images via sequential sampling. Unlike eye-movement attention tracking (emAT), a participant clicks the location in the image that he wants to see (a form of *mouse-click attention tracking* (mcAT)). Immediately after that, he selects the class(es) that he predicts the object might belong to based on his observations so far. Thus, at each sampling episode, our data consists of the image location selected, class label(s) predicted, and time taken since last episode by the participant. After each image, the participant receives a reward based on his performance (accuracy and efficiency).

### Advantages of mcAT over emAT for handwritten numeral/alphabet recognition

(1) emAT contains significant intra- and inter-personal variability in fixation location, especially for static stimuli (images)^8,9^. So a large amount of eye fixation data is needed to reach statistically significant conclusions. mcAT is not susceptible to some of the sources of technical noise common to eye-tracking data^10^. (2) Eye movements can result from both voluntary and involuntary mechanisms^11^. To facilitate task-dependent decision-making, we present the participants with adequate time, context and reinforcement signal, which can also be presented to an ML model. (3) The precision and accuracy of emAT data are dependent on the eye-tracker while the same of mcAT are independent of any device. (4) It is a challenge to synchronize one’s eye movements with his class selection. To overcome this, in our case the sampling location and class(es) are selected in the same episode. (5) Finally, our method allows data collection using Amazon Mechanical Turk (MTurk), as in^12,13^, which is cost- and time-effective, and easily reproducible.

### Contributions

We collect an mcAT dataset, called AttentionMNIST, using MTurk from 382 participants, rewarded for accurately and efficiently recognizing handwritten numerals and alphabets (upper and lowercase) from images via sequential sampling. Images from benchmark datasets (MNIST, EMNIST) are presented as stimuli. On average, 169.1 responses per numeral/alphabet class are recorded. Using this dataset, we show the following:

- On average, participants require 4.2, 4.7 and 4.9 samples to recognize a numeral, uppercase and lowercase alphabet, which correspond to only 11.3%, 13.4% and 13.7% of image area respectively. Classification accuracy increases with number of samples.
- A model, presented as the baseline, can predict the class(es) and location a participant will select at the next sampling episode with 74.4% and 67.7% accuracy respectively, both averaged over all samplings and datasets. Class prediction accuracy increases and location prediction accuracy decreases with increase in samples.
- When exposed to the same stimuli and conditions as our participants, a highly-cited reinforcement-based recurrent attention model (RAM)^3^ requires 3.7, 8.5, 7.6 samples to recognize a numeral, uppercase and lowercase alphabet, which correspond to 8.9%, 21.0%, 18.7% of image area respectively. Other attention-based reinforcement models (e.g.,^1,2,4,5,7,14^) can be similarly evaluated in comparison to human performance.

**Figure 1:**
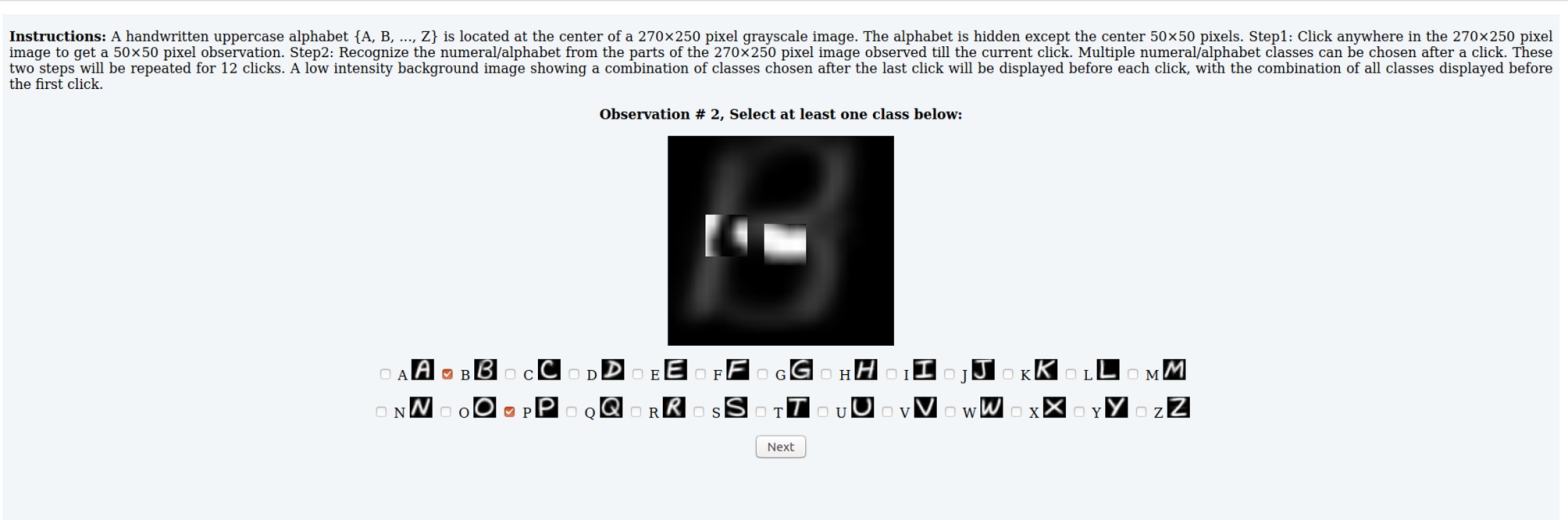
Our MTurk interface as seen by a participant. The second sampling for an EMNIST uppercase alphabet is shown.

## Related work

The temporal sequence of mouse clicks in mcAT is analogous to the eye movement scanpath^10^. mcAT can effectively substitute emAT as they are significantly correlated^10,12,13,15–17^.

Different kinds of stimuli have been used in mcAT studies, such as images of animate and inanimate objects^10^, images of natural scenes^12,13^, static webpages^13^, search page layouts^16^, and two lists of alphanumeric strings for visual comparison^17^. However, mcAT has not been used for handwritten numeral/alphabet classification tasks or evaluation of attention-based classification models.

mcAT studies have used features such as time to contact, relative fixation frequency in areas of interest (AOIs), and relative proportion of subjects that clicked at least once in an AOI^10^, number of fixations per trial, refixations within trials, dwell times, and scanpaths^17^, fixation maps^12,13^, AOI and information flow pattern^16^. The sequence of time-stamped click locations and predicted class labels constitute the raw data necessary to evaluate the efficiency and accuracy of attention-based models or humans in classification tasks. Different features can be derived from this data.

Our mcAT dataset, with multiple benefits over eye-tracking data, fills a crucial gap in attention-based models research in AI, ML, and other areas. Our dataset will allow attention-based models to be evaluated in comparison to human performance. Among other things, this will facilitate the development of efficient and real-time optical character recognition systems that have wide usage in practice (see for example^18–20^). Principles guiding visual fixations can be hypothesized and tested using our dataset. The successful principles can be carried over to develop systems for real-world visual recognition tasks where efficiency is a key concern, such as in autonomous driving.

## Data

Our data consists of a sequence of *T* episodes for each participant. The data from each episode consists of: (1) the location in the image clicked by the participant (one click in image per episode), (2) the class(es) selected by the participant, and (3) the time taken by the participant to register the current sample (i.e. the time elapsed between the last and current clicks in the image). This section will explicate our data collection process that includes stimuli selection, participants, visual task, performance scoring, and data filtering.

### Stimuli selection

Stimuli are selected from images in two benchmark datasets:

1. **MNIST**^21^ dataset consists of $70,\!000$ labeled images ($28\!\times \!28$ pixels) of 10 handwritten numerals $\{0,1,\ldots ,9\}$.
2. **EMNIST**^22^ dataset consists of $145,\!600$ images ($28\!\times \!28$ pixels) of handwritten English alphabets in uppercase and lowercase, forming a balanced class. All images are labeled with one of 26 classes $\{a,b,\ldots ,z\}$. However, uppercase or lowercase label is not associated with any image.

From each category, we select 15 well-formed numerals from MNIST and 15 well-formed alphabets each from EMNIST uppercase and EMNIST lowercase datasets. A well-formed numeral or alphabet is one that is similar to the norm of its class. Thus, we present stimuli from a set of $15(10+26+26) = 930$ unique images, with 15 images belonging to each of the 62 classes.

The well-formed 930 images are selected as follows:

**Step 1**: Normalize each image using min-max to scale the intensity between 0 and 1.
**Step 2**: Label well-formed EMNIST images as uppercase or lowercase. For each alphabet class, a well-formed alphabet from both uppercase and lowercase images is manually selected and labeled. The cosine similarity of all images belonging to that class with the two labeled images is computed. The images that are above the cosine similarity threshold (empirically chosen as 0.8) are assigned the uppercase or lowercase label.
**Step 3**: Compute the mean of the images belonging to each class. The mean image of a class constitutes its norm. An image is eligible to be a stimulus if its cosine similarity with the mean image of its class is greater than an empirically-determined threshold (0.7 for MNIST, 0.75 for EMNIST).
**Step 4**: Among the eligible images, 15 images from each class are selected manually based on how well-formed they are.

Each image, originally $28\!\times \!28$ pixels, is reduced to $27\!\times \!25$ by removing the pixels near the boundaries as they have no intensity variation. The mean of these 15 images is computed for each of the 62 classes. We denote these mean images as $I_1, I_2, \ldots , I_n$ for *n* classes in each dataset.

### Participants

A total of 382 distinct adult individuals participated in our study. No selection criteria were used. A participant could respond to multiple images. For each of the 62 classes, an average of 169.1 responses were recorded.

### Visual task

The MTurk interface for our visual task is shown in Fig. 1. A canvas of size $270 \!\times \!250$ displays a low-intensity background image at all time. The background and stimulus images are upsampled ten times to $270\!\times \!250$. The center of the canvas is aligned with the center of the images.

*Background* Initially, the background is the mean of all images in the dataset from which the stimulus is drawn. After the first episode, the background is the mean of all images from the set of classes selected by the participant in the last episode. In the real world, the context for location, size and orientation of a numeral or alphabet is obtained from the writing in its neighborhood, which is missing here. When our experiments were conducted with a blank background, the participants often sampled locations of the image that do not contain any part of the object. This behavior was contained by presenting the mean image of the selected class(es) in a low-intensity background and reducing the size of all MNIST and EMNIST images from $28\!\times \!28$ pixels to $27\!\times \!25$.

Each time the participant selects a location in the canvas by clicking on it, a $50\!\times \!50$ pixel patch centered at that location from the stimulus image is revealed. A patch once revealed continues to be displayed till the final episode.

A participant’s task consists of three steps at each episode *t* ($t=1,\ldots ,T$):

**Step 1**: Click anywhere in the $270\!\times \!250$ canvas to reveal the patch he wants to sample. Only the first click is accepted.
**Step 2**: Recognize the numeral/alphabet from all the samples observed so far. The participant can select multiple classes and will have to choose at least one class from the list of classes shown below the canvas.
**Step 3**: Click “Next” at the bottom of the screen to proceed.

In order to infer the class accurately and quickly, the participant will have to choose the locations judiciously given his observations till the current episode. There is no time limit for an episode. However, we limit the total time for *T* episodes of an image to six minutes. We choose $T=12$ as highly-cited works on attention-based handwriting recognition or generation have used fewer than 12 glimpses (e.g., RAM^3^ could recognize MNIST numerals within 7 glimpses, DRAW^23^ could generate MNIST numerals within 11 glimpses), and humans can recognize handwritten numerals and alphabets in much fewer than 12 glimpses.

### Performance scoring

A score is assigned to the participant based on his accuracy and efficiency in terms of the number of samples observed. Let $c_t$ be the set of classes he chose at any episode *t*. Then, his score at *t* is:

1 $$\begin{aligned} P_t = {\left\{ \begin{array}{ll} \frac{1}{|c_t|}, &{} \text {if correct class} \in c_t\\ 0, &{} \text {otherwise} \end{array}\right. } \end{aligned}$$

where |.| denotes the cardinality of a set. Total score awarded in *T* episodes is: $h = \sum _{t=1}^{T}P_t$. Therefore, the maximum one can score in *T* episodes is *T* if he always chooses only the correct class. The minimum one can score in *T* episodes is zero if he always chooses a set of classes that does not include the correct class. So, $0\le h\le T$.

Sooner a participant selects the correct class, the higher his score will be. Thus, this scoring mechanism takes into account recognition accuracy and sampling efficiency. Trying to maximize score by choosing only one class from the very first episode will be risky as a score of zero will be awarded if it is not the correct class, whereas a score greater than zero will be awarded if the participant chooses multiple classes (even all classes) that include the correct class. This will motivate the participant to respond based on the probable classes in his mind at any episode. The score awarded at each episode is disclosed only upon completion of *T* episodes to refrain from providing any hint to the participant. In MTurk, the remuneration received by a participant for an image is proportional to his total score, *h*.

### Data filtering

If a participant’s score at the final (i.e. *T*-th) episode for a stimulus image is zero, his data recorded for that image is discarded. The data is also discarded if a participant leaves the task incomplete. With this selection criteria, we obtained responses on 1736 stimuli from MNIST, 4431 stimuli from EMNIST uppercase, and 4315 stimuli from EMNIST lowercase; that is, 169.1 responses per class on average.

## Models and methods for utilizing data

In this section, we illustrate the utility of the collected data by: (4.1) providing a baseline model for predicting the behavior of a participant, and (4.2) showing how an existing attention-based reinforcement model can be compared to human numeral/alphabet recognition performance.

### Baseline for behavior prediction

Behavior at any episode *t* consists of location selection and class selection. Since a sample contains different amounts of information for different observers, or even for the same observer at different times^9^, behavior prediction of each participant is a difficult problem. Let *n* be the number of classes in a dataset, $\eta _{t}$ be the singleton set containing the true class for the stimulus image at *t*, $c_{t}$ be the set of classes and $l_{t}$ be the location selected by a participant at *t*, $o_{t}$ be his observation at *t*, and $1\!\!:\!t$ denotes the sequence $1,2,\ldots ,t$. Till any *t*, the observations of a participant are $o_{1:t}$ and the locations he selected are $l_{1:t}$.

We formulate the problem of a participant’s behavior prediction as follows:

*Class prediction* Estimate the probability of $i\!\in \!c_{t}$ ($i=1,2,\ldots ,n$) given his $o_{1:t}$ and $l_{1:t}$, i.e. $P(i\in c_t|o_{1:t},l_{1:t})$.

*Location prediction* Estimate the probability of $l_{t+1}$ given his $o_{1:t}$, $l_{1:t}$ and $c_t$, i.e. $P(l_{t+1}|o_{1:t},l_{1:t},c_t)$.

#### Class prediction

To predict the class a participant will choose at episode *t*, we compute the probability that the image stimulus at *t* belongs to class *i* given the participant’s selected locations $l_{1:t}$ and the corresponding observations $o_{1:t}$, as follows:

2 $$\begin{aligned} P(i|o_{1:t},l_{1:t}) = \frac{\frac{I'}{\Vert I'\Vert }\cdot \frac{I_i}{\Vert I_i\Vert }}{\sum _{j\in {\{1,\ldots ,n\}}}\frac{I'}{\Vert I'\Vert }\cdot \frac{I_j}{\Vert I_j\Vert }} \end{aligned}$$

where $I_i$ is the mean of the stimuli images ($27\!\times \!25$) belonging to class *i*, $I'$ is a $27\!\times \!25$ image containing $o_{1:t}$ at $l_{1:t}$, $\cdot$ denotes scalar product, and $\Vert .\Vert$ denotes Euclidean norm. All pixel intensities are non-negative.

At any episode *t*, the *k* highest probable classes from the belief distribution $P(i|o_{1:t},l_{1:t})$ constitute the set of classes, $\hat{c}_t$, predicted by our model, where $k=|c_t|$.

The classification accuracy is measured using the Jaccard index (JI). JI measures the similarity between two sets, *X* and *Y*, as: $J(X,Y) = |X\cap Y|/|X\cup Y|$. JI is bounded between 0 and 1; if $X=Y$, $J(X,Y)=1$. At any episode *t*, the classification accuracy of a participant is $J(\eta _t,c_t)$ while that of our model is $J(\eta _t,\hat{c}_t)$. Due to its denominator, JI penalizes more as the number of elements in the predicted set ($c_t$ or $\hat{c}_t$) that are not in $\eta _t$ increases, which is a desirable property for our case. The similarity between a participant’s and our model’s classification is measured by $J(c_t,\hat{c}_t)$.

Our model is also evaluated in terms of class selection and rejection accuracy with respect to each participant. Let $s_t=c_t-c_{t-1}$ be the set of new classes selected and $r_t=c_{t-1}-c_t$ be the set of classes rejected by a participant at *t*. Similarly, $\hat{s}_t=\hat{c}_t-c_{t-1}$ be the set of new classes selected and $\hat{r}_t=c_{t-1}-\hat{c}_t$ be the set of classes rejected by our model at *t*. Then the model’s class selection and rejection can be compared to a participant’s by $J(s_t,\hat{s}_t)$ when $|s_t|>0$ and $J(r_t,\hat{r}_t)$ when $|r_t|>0$, respectively.

#### Location prediction

**Figure 2:**
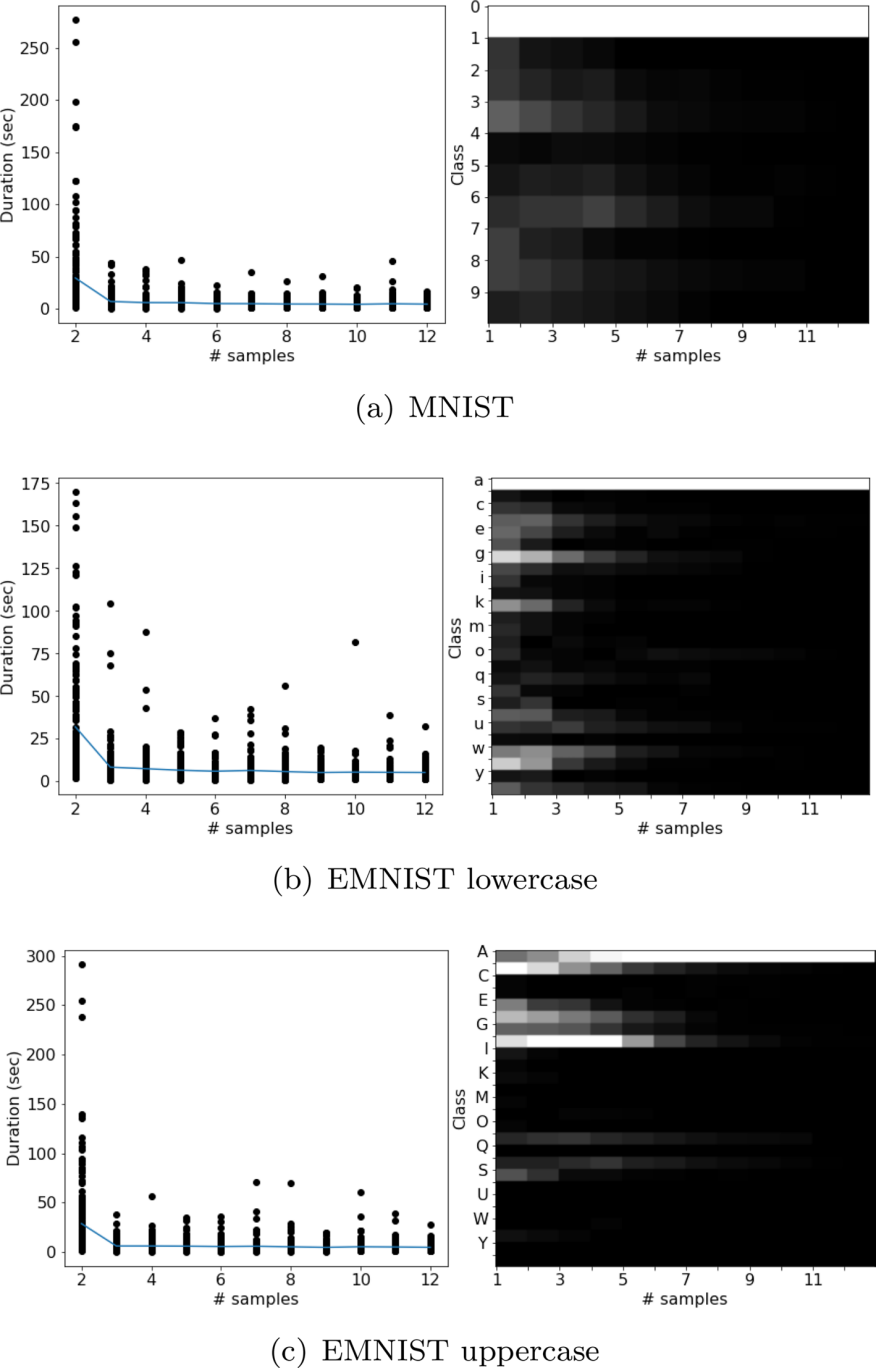
Duration and class distribution over all participants and stimuli belonging to categories ‘0’, ‘a’ and ‘A’.

*Hypothesis* Ideally, the belief distribution over all classes should be unimodal (i.e., one peak only) and a thin Gaussian (i.e., small standard deviation) in shape indicating a participant is confident about the class (state) of the stimulus (environment). However, as evident from our data (ref. Fig. 2), a participant is often confused between multiple classes, especially during the initial few episodes. In these cases, his belief distribution has multiple peaks or is a fat Gaussian. We hypothesize, a participant’s goal is to converge to a unimodal and thin Gaussian, to achieve which he selectively samples locations that reduce the probability of all classes except one. This hypothesis leads to minimization of uncertainty over the classes (environmental states) which is a well-known principle guiding action^24^, including eye movements^25^.

The observations at certain locations in a stimulus image can discriminate between certain classes. The observation at a location *l* might indicate that the numeral/alphabet belongs to a class *i* and not to a class *j*. Such locations are more salient than others in achieving a participant’s goal. To sample such locations, a saliency map, $D_{ij}$, is computed such that if *l* is salient, the observation at *l* is an evidence to increase the probability of class *i* and decrease that of *j*.

Mathematically, $D_{ij} = \mathcal {N}(.,\sigma ) * g(.)$, where $*$ is the convolution operator, *g*(.) is a saliency scoring function, and $\mathcal {N}(.,\sigma )$ is a 5$\times$5 Gaussian kernel with standard deviation $\sigma = 6$ to smooth the saliency scores. We denote the set of all saliency maps as $\text {D}=\{D_{ij}:i,j\in \{1,2,\ldots ,n\}, i\ne j\}$. A location *l* in a stimulus image is salient for class *i* with respect to class *j* if $D_{ij}(l)>\theta$, where the threshold $\theta = 0.5\times \max (\text {D})$ is an empirically determined scalar quantity.

We consider two asymmetric metrics, Kullback-Leibler (KL) divergence and difference, as candidates for the function *g*.

*KL divergence* Given two normalized mean images, $$I_i$$ and $$I_j$$, the KL divergence $$ KL (I_i,I_j)$$ measures the loss of information when $$I_j$$ is used to approximate $$I_i$$. This is calculated for each pixel *k* as^26^: $$ KL (I_{i,k},I_{j,k}) = I_{i,k} \log \Big (\delta + \frac{I_{i,k} }{I_{j,k} +\delta }\Big )$$, where $$I_{j,k}$$ is the intensity of the $$k^{th}$$ pixel of $$I_{j}$$, and $$\delta $$ is a regularization constant. When $$I_{i,k}=I_{j,k}$$, $$ KL (I_{i,k},I_{j,k})\rightarrow 0$$.

*Difference* Given two normalized mean images, $$I_{i}$$ and $$I_{j}$$, the difference for each pixel *k* is: $$ Diff (I_{i,k}, I_{j,k} ) = I_{i,k} - I_{j,k} $$. When $$I_{i,k}=I_{j,k}$$, $$ Diff (I_{i,k},I_{j,k})=0$$.

A participant is uncertain regarding the set of classes, $c_t$, he selected at the current episode. Hence, for location prediction, we consider only those saliency maps in $\text {D}$ that involve the classes in $c_t$. A location is predicted if it is salient based on these saliency maps and was never selected by the participant. Thus, given $o_{1:t}$, $l_{1:t}$ and $c_t$, the location $l_{t+1}$ is predicted as follows:

3 $$\begin{aligned}{}&\text {D}' = \{D_{ij}:D_{ij}\in \text {D}, i\in c_t ~\text {or}~ j\in c_t\}\nonumber \\&\Gamma = \{\langle \hat{l},i,j \rangle : \hat{l}\notin l_{1:t}, D_{ij}(\hat{l})>\theta , D_{ij}\in \text {D}'\} \end{aligned}$$

where $\Gamma$ is the set of 3-tuples containing the predicted location $\hat{l}$, the class it is salient for (*i*), and with respect to which class (*j*). The location is predicted correctly if there exists a $\langle \hat{l},i,j\rangle \in \Gamma$ such that $\Vert \hat{l}-l_{t+1}\Vert <\epsilon$, $i\in c_{t+1}$ and $j\notin c_{t+1}$, where $\epsilon$ is the maximum Euclidean distance between the center pixel and any pixel in an observation patch. The pseudo code for location prediction is shown in Algorithm 1. Detailed explanation of the pseudo code is included in Section S1 of supplemental material. (The probability distribution, $P(l_{t+1}|o_{1:t},l_{1:t},c_t)$, may be computed by assuming the saliency score of locations not in $\Gamma$ to be zero, and then normalizing the saliency score of all locations to sum to unity. However, this probability has not been used, as Eq. (3) is sufficient for the purposes of this paper.)
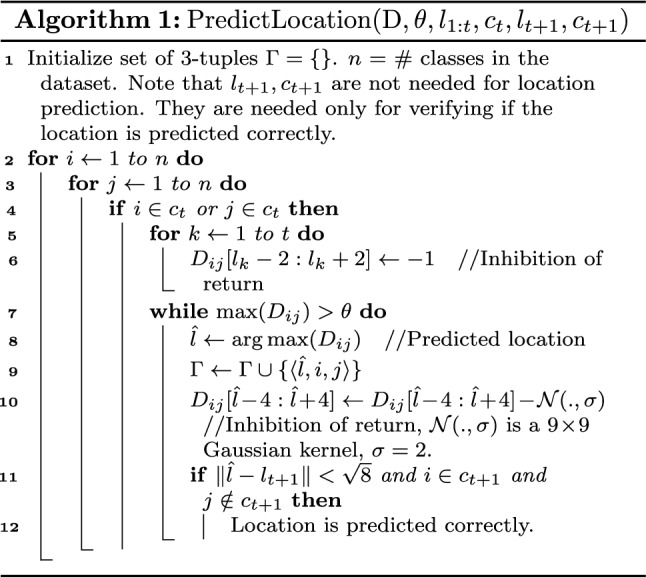


### Evaluation of attention-based models

As a representative of attention-based models, we consider the highly-cited recurrent attention model (RAM)^3^ that reports experimental results on the MNIST dataset. This reinforcement model sequentially samples an image and decides where to sample next at each sampling instant, making it appropriate for evaluation using the collected data.

**RAM** classifies images using a sequence of glimpses. The next location is chosen stochastically from a distribution parameterized by a location network. The model is trained end-to-end by maximizing the following objective^3^:

4 $$\begin{aligned} \frac{1}{M} \sum _{i=1}^{M} \sum _{t=1}^{T}\Delta _{\theta }\log \pi (u_{t}^{i}|x_{1:t}^i;\theta )(R^{i}_{t}-b_{t}) \end{aligned}$$

where *M* is the number of episodes, *T* is the number of observations, $x_{1:t}^{i}$ are the interaction sequences obtained by running the current agent till *i* episodes, $u_{t}^{i}$ is the current action, $\theta$ is the set of trainable parameters, $R^{i}_{t}$ is the cumulative reward, $b_t$ is a baseline, and $\pi (u_{t}^{i}|x_{1:t}^i;\theta )$ is the policy. RAM’s behavior may be compared with the participants’ by comparing the fixation maps obtained from the sequence of locations predicted by RAM and those chosen by the participants. A fixation map is computed by assigning each location a value equal to the frequency of its selection, and then normalizing those values to create a distribution over all locations.

#### Metrics for comparing fixation maps

For metrics comparing two fixation maps, *P* and *Q*, we closely follow^26^. We use three distribution-based metrics: KL divergence (KL), Pearson correlation coefficient (CC), and Similarity (SIM), to compare the distribution of sampling locations from a model with that from the participants as recorded in the collected data.

- **KL** (defined earlier) is highly sensitive to zero values.
- **CC** can evaluate the linear relationship between two maps as^26^: $CC (P,Q) = \frac{\sigma (P,Q)}{\sigma (P)\sigma (Q)}$, where $\sigma$ is the variance or covariance. Since CC is symmetric, it fails to infer whether differences between fixation maps are due to false positives or false negatives.
- **SIM** is measured as^26^: $SIM (P,Q) = \sum _{k} \min (P_{k},Q_{k})$, where $\sum _{k}P_{k} = \sum _{k}Q_{k} = 1$. Like CC, SIM is symmetric and inherits the same drawback. Also, SIM is very sensitive to missing values, and penalizes predictions that fail to account for the ground truth density.

**Figure 3:**
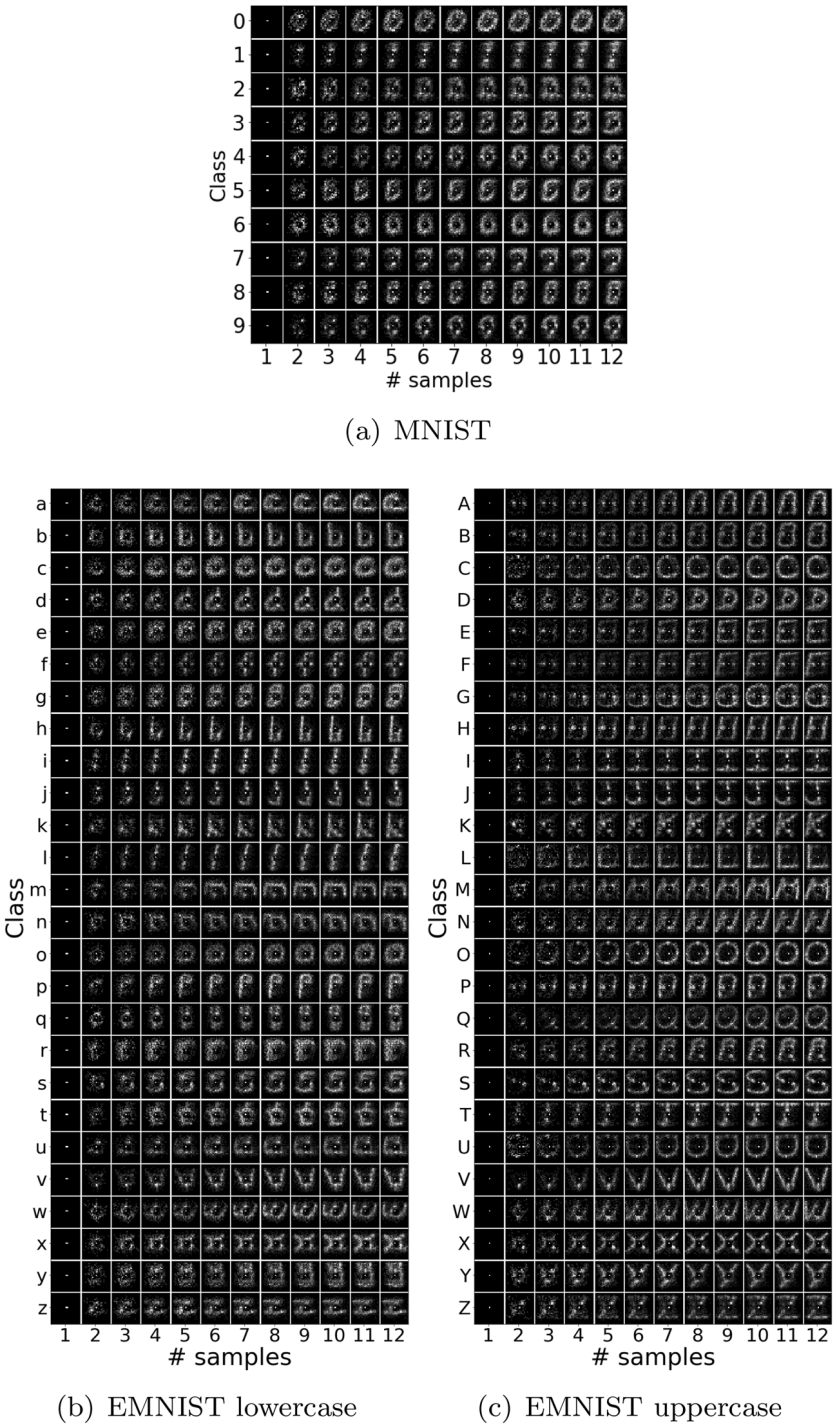
Distribution of sampling locations over all participants for each numeral/alphabet class and each sampling episode. Each row corresponds to a class, each column corresponds to a sampling episode which increases from left to right.

**Figure 4:**
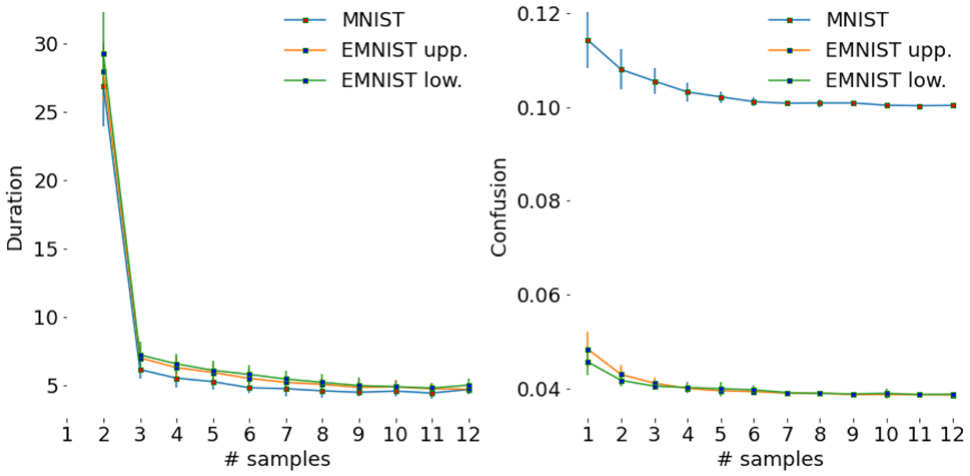
(Left) Errorbar plot of time difference (seconds) between consecutive samples averaged over all classes. That is, value shown at sampling episode *t* is the time elapsed between a participant’s clicks in image at $t-1$ and *t*. (Right) Errorbar plot of confusion averaged over all classes at each episode. Errorbars indicate std. dev.

### Human and Animal Research

The Institutional Review Board at the University of Memphis has determined that this study does not meet the Office of Human Subjects Research Protections definition of human subjects research and 45 CFR part 46 does not apply. Hence, this study does not require IRB approval nor review.

## Experimental results

### Data analysis

**Figure 5:**
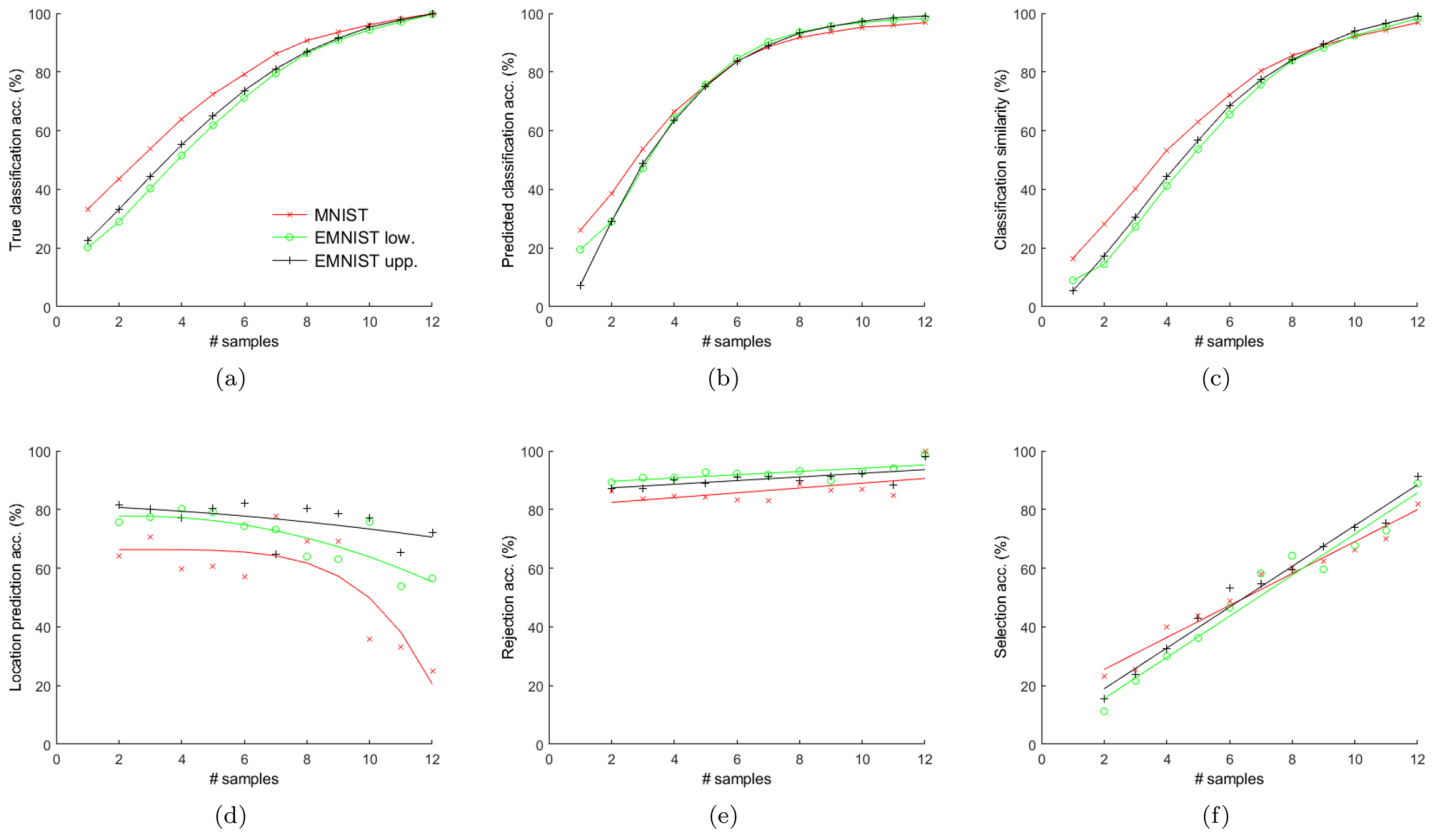
Evaluation of our baseline model (ref. “Baseline for behavior prediction” Section). (**a**) Classification accuracy (acc.) of the participants and (**b**) that of our baseline model with actual labels as ground truth. (**c**) Classification similarity ($J(c_t,\hat{c}_t)$), (**d**) location prediction accuracy, (**e**) class rejection accuracy and (**f**) class selection accuracy of our baseline model with participants’ data as ground truth. See “Behavior prediction” section for details.

The collected data can be visualized in terms of the sequence of distribution of selected locations (Fig. 3), selected classes (Fig. 2), and duration between consecutive episodes (Fig. 2). These distributions are very similar for the three datasets.

For any numeral or alphabet, the distribution of selected locations after the final episode resembles the distribution of pixel intensities of its class from the dataset. However, the sequence of locations selected is stochastic in nature.

The class distribution indicates confusion between categories with similar structures at the initial few episodes when the participants choose multiple classes. This confusion reduces with more sampling. There is a significant positive correlation between degree of confusion (# selected classes/total # classes) and sampling duration (see Fig. 4). If the number of selected classes is high (low), the duration between consecutive episodes is high (low).

The CC of the sequence of locations selected by a participant for a class is not significant (Table 1). This is expected due to inter-subject variability in sampling static images.

**Table 1:** Average Pearson correlation coefficient (corr.) for fixation sequences for the same class. For any fixation, distance is Euclidean and direction is measured as the polar angle with respect to the center of stimuli as origin. Std. dev. are included in parenthesis.

Metric	MNIST	EMNIST upp.	EMNIST low.
Distance corr.	0.34 (0.21)	0.42 (0.22)	0.33 (0.21)
Direction corr.	0.27 (0.19)	0.28 (0.21)	0.29 (0.2)

The average number of samplings required by a participant to accurately predict a class is quite low. On average, it takes 4.2, 4.7, 4.9 samples corresponding to 36, 44.1, 48.1 seconds to accurately classify MNIST, EMNIST uppercase and lowercase images respectively. The participants on average viewed only $11.3\%$, $13.4\%$, $13.7\%$ of image area for classifying a numeral, uppercase and lowercase alphabet image accurately (see Fig. S2 in supplemental material). These results highlight the efficiency of the human visual reasoning system, albeit at a lower resolution than eye tracking data but with less noise and variability. These empirical results may be useful for designing attention-based models for real-world applications.

### Behavior prediction

In this section, the performance of our baseline model is evaluated in terms of how accurately it can predict each participant’s location and class selection. Since our experimental results using the two saliency scoring functions, KL divergence and difference, are quite similar, results are reported using difference only, unless otherwise stated.

#### Class prediction

The class prediction and its accuracy evaluation methods are described in “Class prediction” section. The class prediction accuracy, shown in Fig. 5, is computed over all classes for all samplings. The mean class prediction accuracy over all samplings and datasets is 74.4% (std. dev. 26.5).

Figures 5a, b show that the set of classes selected by the participants and by our baseline model (Eq. 2) are quite inaccurate at the initial episodes and improves with increase in samples. Figure 5c shows that, during the initial episodes, these two sets, $c_t$ and $\hat{c}_t$, are quite dissimilar; similarity increases with increase in samples. The same applies to new class selections (ref. Fig. 5f). However, class rejections are similar at the initial episodes; similarity increases further with more samples (ref. Fig. 5e). Since $\displaystyle J(s_t,\hat{s}_t)=\frac{|(c_t\cap \hat{c}_t)-c_{t-1}|}{|(c_t\cup \hat{c}_t)-c_{t-1}|}$ and $\displaystyle J(r_t,\hat{r}_t)=\frac{|c_{t-1}-(c_t\cup \hat{c}_t)|}{|c_{t-1}-(c_t\cap \hat{c}_t)|}$, it can be inferred from Fig. 5e, f that at the initial episodes, the intersection between $c_{t-1}$ and $c_t\cup \hat{c}_t$ is small, indicating that initially the participants and our baseline model make many changes in their class selection between consecutive episodes. Therefore, initially, the class selection process is highly stochastic.

While there are some dissimilarities between the participants’ and our model’s class prediction during the initial episodes, the behaviors become increasingly similar with more samples. During the first few (typically 4 to 7) episodes, highly salient parts of a stimulus are revealed. This helps to select only the correct class in the later samplings, which increases the prediction accuracy. Since there are many classes whose mean templates match the observed parts of the stimulus during the initial few episodes, the class selection process is significantly more stochastic, leading to low classification accuracy from the participants as well as our model.

#### Location prediction

Our baseline model’s (Eq. 3) location prediction accuracy, averaged over all samplings and datasets, is 67.7% (std. dev. 14.1) (ref. Fig. 5d). The trend of this prediction accuracy is opposite to that of class prediction accuracy. However, the explanation remains the same. Location prediction accuracy is high during the initial samplings because during these episodes, the highly salient locations are selected, leaving the less salient locations to be selected in the later episodes. Since there are many locations with low saliency, their selection process is highly stochastic and hence difficult to predict, leading to a decrease in prediction accuracy with increase in samplings. The decreasing trend is unique for each dataset (ref. Fig. 5d) as the number of classes and the number of highly salient locations useful for discrimination vary between datasets. Lower the number of classes and highly salient discriminative locations, faster will be the decrease in location prediction accuracy with increase in samplings.

### Evaluation of RAM

For each class and sampling, the fixation maps from RAM (we used the RAM implementation from github.com/hehefan/Recurrent-Attention-Model) and the collected data for the same stimuli presented in MTurk are compared. For a fair comparison with the participants, in RAM we fixed the sequence length at $T=12$, the first sampling location at the image center, the input observation to a $5\!\times \!5$ patch with the selected location as its center, and modified the reward function by Eq. (1). The cumulative reward, $R_t$ in Eq. (4,) is replaced by the cumulative score $\sum _{\tau =1}^{t} P_{\tau }$ obtained from Eq. (1). As a participant can select multiple classes at any episode, for the RAM model, instead of predicting a single class based on highest probability, we consider the mean probability over all classes as a threshold and predict the set of classes $c_t$ with probabilities greater than the threshold. This $c_t$ is used for calculating the score using Eq. (1).

Under these conditions, RAM requires 3.7, 8.5, 7.6 samples to recognize MNIST numerals, uppercase and lowercase EMNIST alphabets, which correspond to 8.9%, 21.0%, 18.7% of image area respectively. Thus, in comparison to our participants (ref. “Data analysis” section), RAM is less efficient. See Table 2.

**Table 2:** Comparison of efficiency between our participants and the RAM model in terms of the average number of samples required to recognize a numeral/alphabet. Percentage of image area observed is included in parenthesis.

	MNIST	EMNIST upp.	EMNIST low.
Participants	4.2 (11.3)	4.7 (13.4)	4.9 (13.7)
RAM	3.7 (8.9)	8.5 (21.0)	7.6 (18.7)

Results from comparing the fixation maps from RAM and the collected data are shown in Table 3. KL is higher due to its sensitivity to zero values. This implies several locations are sampled by the participants but not by RAM. These experiments can be used as a baseline for evaluating locations sampled by an attention model.

**Table 3:** Evaluation of fixation maps from RAM for the stimuli presented in the MTurk experiments, averaged over all classes and samplings. Std. dev. are included in parenthesis.

Metric	MNIST	EMNIST upp.	EMNIST low.
KL	22.50 (7.48)	22.96 (7.24)	22.23 (7.16)
CC	0.01 (0.00)	0.01 (0.00)	0.01 (0.00)
SIM	0.17 (0.09)	0.16 (0.07)	0.18 (0.09)

## Discussions

The mcAT paradigm, as used in this paper, has certain points of difference from those that primarily rely on eye movements and gazes to study the mechanisms of object recognition. In the latter, salient parts of the scene attract attention first, followed by saccadic eye movements directing the eye gaze to the salient locations^27^. Gaze is driven by bottom-up and top-down signals which, together with salience information, form priority maps that guide eye movements for object recognition. Since participants in the present study looked at the static images under free-viewing conditions and with ample time at hand (six minutes for *T*=12 samplings), they likely engaged in a series of saccadic eye movements or visual reasoning^28^ to explore the image before clicking on an AOI. These eye movements could have been captured in emAT (using an eye-tracker) but not in mcAT. However, these eye movements are affected by mind wandering. While mcAT is also affected by mind wandering^29^, the effect may be reduced whenever the participants responded after visual reasoning.

Since eye movements in response to a stimulus are influenced by the task at hand^30^, the participants’ eye movement patterns were likely influenced by the assigned three-step task at each sampling (ref. “Visual task” section). If an eye-tracker was used, the participants’ eye movements to explore the sample would have been intermixed with eye movements to click their chosen classes, which would have complicated the interpretation of the visual exploration of the sample. Clicking the class(es) is a necessary step as it reveals, albeit introspectively, the predicted class(es) in the mind of a participant.

It is likely that the gazes immediately before and after the AOI selection—perhaps also aided by fixational eye movements^31^—contributed the most towards the numeral/alphabet recognition. Indeed, we surmise that participants selected diagnostic areas of the image to distinguish between classes, and those areas likely contain a mixture of bottom-up (e.g., visual contrast) and top-down (numeral/alphabet template) diagnostic information. This is consistent with our finding that participants quickly (within 5 samples on average) distinguished between stimulus classes ostensibly by selecting diagnostic patches.

## Conclusions

We introduced an mcAT dataset for recognizing handwritten numerals and alphabets via sequential sampling. The data is collected from 382 participants presented with images selected from benchmark datasets (MNIST, EMNIST). On average, 169.1 responses per numeral/alphabet class are recorded. The data is rigorously analyzed to reveal the efficiency of human visual recognition. The participants observed only 12.8% of an image for recognition. We proposed a baseline model to predict the location and class(es) a participant would select at the next sampling. We showed how our experimental conditions and data may be used to evaluate an attention-based reinforcement model in comparison to human performance. This mcAT dataset, with multiple benefits over eye-tracking data, fills a crucial gap in attention-based models research in AI, ML, and other areas.

## Supplementary Information

Supplementary Information.

## Data Availability

The dataset collected, used and analyzed during the current study is available from the corresponding author on reasonable request.
