# Supplementary material for: AttentionMNIST: a mouse-click attention tracking dataset for handwritten numeral and alphabet recognition
Source: Sci Rep. 2023 Feb 27;13:3305. doi: 10.1038/s41598-023-29880-7 (PMC9971057; doi:10.1038/s41598-023-29880-7)
Supplement: Supplementary file 1 — Supplementary Information. [file 41598_2023_29880_MOESM1_ESM.pdf]

Supplemental Material

S1 Location prediction pseudocode

Our hypothesis for location prediction (ref. Section 4.1.2) is implemented as Algorithm 1. According to our hypothesis, a participant is minimizing uncertainty from his current selection of classes, c_t. Initially, the saliency maps that involve the classes in c_t are selected (ref. line 4 in Algorithm 1).

To conform with our MTurk experimental setup, inhibition-of-return is utilized in the baseline model. That is, a location cannot be resampled if it has already been sampled (ref. line 6 in Algorithm 1). This is achieved by assigning a negative value to a 5×5 patch centering the previously sampled location.

A location(s) in each saliency map with saliency value greater than a threshold θ is selected (ref. line 9 in Algorithm 1) which is added to the set of 3-tuples Γ along with the classes involved in the current saliency map (ref. line 10 in Algorithm 1) .

In order to ensure this location is not resampled within the loop at the same episode, inhibition-of-return is applied (ref. line 11 in Algorithm 1) before selecting the next location from that saliency map. This is done by lowering the saliency values from a 9×9 patch centering that location. Setting a global threshold allows comparison of all the saliency maps and allows selection of variable number of locations from each saliency map. The average number of locations selected from a saliency map is less than three in all cases (see Fig. S1).

If the participant selects a location which lies close to the predicted salient location $\hat{l} = \arg\max(D_{ij})$, and he selects class i and does not select class j, the predicted location is considered to be correct. The Euclidean distance between the predicted location and participant's selected location has to be less than $\sqrt{8}$, which is the maximum distance between the center pixel and any other pixel of a 5×5 patch.

Note that the Gaussian kernel is used in the calculation of saliency map D_{ij} and in line 10 of Algorithm 1. The Gaussian function ensures that the score (probability) decreases exponentially with increase in distance from the mean. We exploit this property of Gaussian function to assign a soft score to each pixel in the neighborhood of previously selected locations such that the model can choose the *best* location far from all previously selected locations. If a hard score is assigned to each pixel, there will be cases where multiple pixels will be equally good even though some of them are near previously selected locations while the rest are far. Such situations are minimized by using a soft score.

S2 Experimental Results: Data analysis

Detailed analysis of the participants' class selection for each dataset is shown in Fig. S2.

S3 Data samples

See Table S1 for samples from our dataset. The entire dataset collected, used and analyzed during the current study is available from the corresponding author on reasonable request.

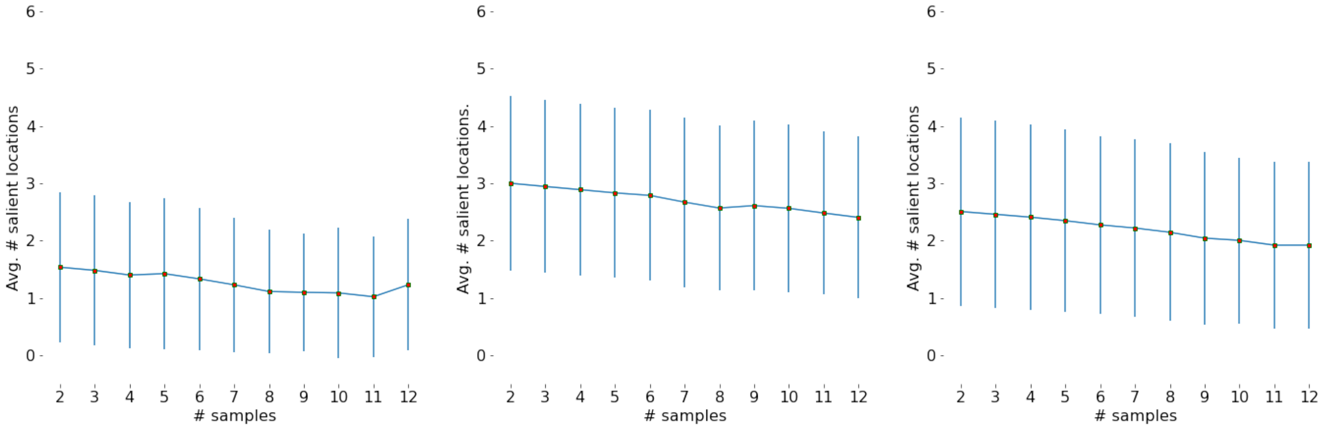

(a) MNIST (b) EMNIST uppercase (c) EMNIST lowercase

Figure S1: Errorbar plot showing the average number of salient locations selected from a saliency map for each sampling. Errorbars indicate std. dev.

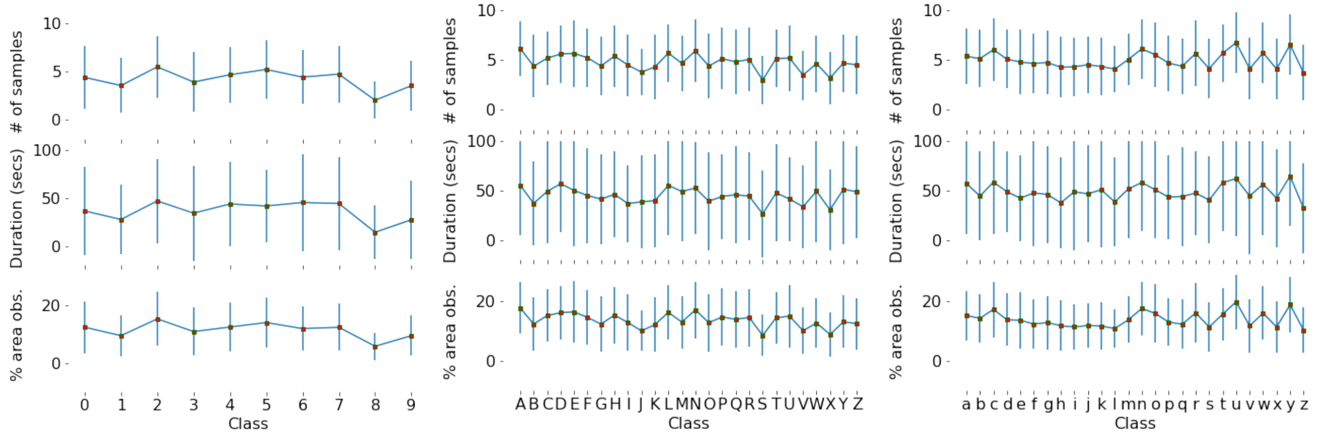

(a) MNIST (b) EMNIST uppercase (c) EMNIST lowercase

Figure S2: Minimum number of samples, corresponding time spent, and proportion of image area observed by the participants after which only the correct class is selected till the last sampling episode. Errorbars indicate std. dev.

Table S1: Two samples from our dataset. One stimulus is from MNIST, the other from EMNIST-uppercase.

Stimulus MNIST (Class)	Observ-ation sequence	Sampled locations (x, y)	Duration between samples (sec)	Class(es) selected	Stimulus EMNIST (Class)	Observ-ation sequence	Sampled locations (x, y)	Duration between samples (sec)	Class(es) selected
	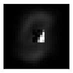	(14, 12)	-	3		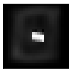	(14, 12)	-	G, U
	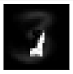	(13, 17)	29.35	3		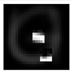	(17, 19)	9.37	G, O
	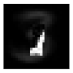	(14, 10)	8.52	3		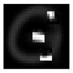	(20, 5)	12.79	G, O, P
	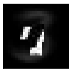	(9, 11)	5.78	4		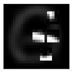	(20, 12)	21.77	G, O, P
	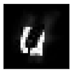	(7, 16)	7.54	4		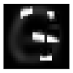	(9, 3)	11.47	G, O, P
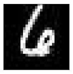<br>6	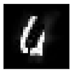	(12, 7)	4.66	4	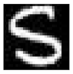<br>S	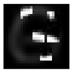	(21, 8)	16.84	G, O
	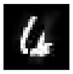	(18, 17)	7.25	6		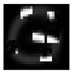	(3, 16)	17.69	G, S
	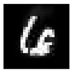	(18, 10)	10.22	6		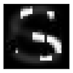	(21, 16)	39.32	S
	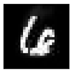	(20, 16)	4.58	6		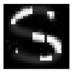	(5, 5)	6.21	S
	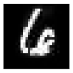	(13, 4)	3.97	6		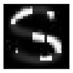	(21, 9)	6.51	S
	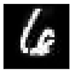	(21, 16)	4.41	6		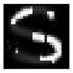	(22, 20)	7.9	S
	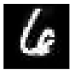	(9, 20)	3.38	6		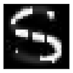	(7, 12)	6.12	S
